# Scope, content and quality of clinical pharmacy practice guidelines: a systematic review

**DOI:** 10.1007/s11096-023-01658-x

**Published:** 2023-11-22

**Authors:** Vibhu Paudyal, Betul Okuyan, Martin Charles Henman, Derek Stewart, Daniela Fialová, Ankie Hazen, Monika Lutters, Anna Oleárová, Anita E. Weidmann, Francesca Wirth, Cathal A. Cadogan, Zachariah Nazar

**Affiliations:** 1 School of Pharmacy, College of Medical and Dental Sciences, Sir Robert Aitken Institute for Medical Research, University of Birmingham, Edgbaston, Birmingham UK; 2 Department of Clinical Pharmacy, Faculty of Pharmacy, Marmara University, Istanbul, Türkiye; 3 Trinity College Dublin, Dublin, Ireland; 4 College of Pharmacy, QU Health, Qatar University, Doha, Qatar; 5 Department of Social and Clinical Pharmacy, Faculty of Pharmacy in Hradec Králové, Charles University, Hradec Králové, Czech Republic; 6 Department of Geriatrics and Gerontology, 1st Faculty of Medicine, Charles University, Prague, Czech Republic; 7 Julius Centre for Health Sciences and Primary Care, University Medical Center Utrecht, Utrecht, The Netherlands; 8 Kantonsspital Aarau, Aarau, Switzerland; 9 Department of Clinical Pharmacology, Bratislava University Hospital, Bratislava, Slovakia; 10 Department of Clinical Pharmacy, Innsbruck University, Innsbruck, Austria; 11 Department of Pharmacy, University of Malta, Msida, Malta; 12 School of Pharmacy and Pharmaceutical Sciences, Trinity College Dublin, Dublin, Ireland

**Keywords:** Clinical pharmacy, Medicines optimisation, Medication review, Practice guidelines

## Abstract

**Background:**

Guidelines for pharmacy practitioners regarding various clinical pharmacy activities have been published in a number of countries. There is a need to review the guidelines and identify the scope of activities covered as a prelude to developing internationally acceptable common guidelines.

**Aim:**

To review the scope of clinical pharmacy guidelines and assess the extent to which these guidelines conform to quality standards as per the AGREE II instrument.

**Method:**

Medline, Embase, Guideline Central, International Pharmaceutical Abstracts, Google Scholar and Google (for grey literature) were searched for the period 2010 to January 2023. Guidelines which focused on any health care setting and any clinical pharmacy activity were included. Data were extracted and quality assessed independently by two reviewers using the English version of the AGREE II instrument.

**Results:**

Thirty-eight guidelines were included, mostly originating from Australia (n = 10), Ireland (n = 8), UK (n = 7) and USA (n = 5). Areas covered included medication reconciliation, medicines optimisation, medication management and transition of care. As per the AGREE II assessment, the highest score was obtained for the scope and purpose domain and the lowest score for rigour of development, mainly due to non-consideration of literature/evidence to inform guideline development.

**Conclusion:**

Clinical pharmacy guidelines development processes need to focus on all quality domains and should take a systematic approach to guideline development. Guidelines need to further emphasise person-centred care and clinical communication. There is a scope to harmonise the guidelines internationally considering the diverse practices, standards and legislations across different geographies.

**Supplementary Information:**

The online version contains supplementary material available at 10.1007/s11096-023-01658-x.

## Impact statements

- A range of clinical pharmacy practice guidelines have been published with a greater focus on medication review and optimisation and less on areas including communication skills and person-centred care.
- Clinical pharmacy guidelines need to focus more on all quality domains such as the use of evidence in guideline development.
- There is a need to develop international best practice guidelines which could be adapted in different countries in the context of national policies and practices, given the resources identified in this study were restricted in scope to a specific country or setting.

## Introduction

The European Society of Clinical Pharmacy (ESCP) published a position paper in 2022 defining the scope of clinical pharmacy [1]. The paper describes clinical pharmacy as the ‘activities and services focused on optimisation of medicines use through practice and research to achieve person-centred and public health goals’ [1]. The extended definition identifies activities covered by clinical pharmacy including services to support roles around selection, administration, and monitoring of medicines by healthcare professionals, patients, and the public [1]. These activities include clinical pharmacy services such as medication counselling, communication, medication review, reconciliation, and optimisation of pharmacotherapy, as well as advanced services, such as pharmacist prescribing [2, 3].

The scope of clinical pharmacy practice may vary across countries and settings guided by established policies and norms. Indeed, a recently published study has indicated that this variation is also reflected in pharmacy education and training offered within European countries [4]. The recent COVID-19 pandemic illustrated such diversity of regulations and roles with reference to pharmacist involvement in COVID-19 vaccinations, with pharmacists’ roles ranging from traditional compounding and preparation of vaccines to vaccine administration and counselling [5]. Countries such as the UK have introduced pharmacist prescribing models which allow pharmacists to prescribe prescription medicines within their areas of competence [6]. Pharmacist involvement in medicines optimisation within general practice (family physician) clinics in the UK National Health Service [7] and the Netherlands [8] are other examples of step change when discussing emerging new clinical pharmacy roles. Medicines optimisation emphasises on pharmacists working as part of the multidisciplinary team to engage with patients to review, prescribe and deprescribe medicines, provide lifestyle and non-medical interventions, improve adherence to and cost-effectiveness of pharmacotherapy and non-pharmacological strategies, and reduce medicines wastage [9]. Such roles have also been described within other countries in Europe and beyond, such as in the USA, Canada, and Australia [10–13].

Despite these variations in practices, many of the activities such as counselling, communication, medication review and reconciliation are common to a wide range of clinical pharmacy services. It is essential that these activities are informed by standards and evidence-based guidelines to support pharmacists and the wider clinical pharmacy team in delivering the best outcomes for patients and the health system. By definition, guidelines refer to ‘systematically developed statements to assist practitioner decisions about appropriate health care for specific clinical circumstances’ [14]. They help to improve and standardise quality of care and should ideally be developed based on current evidence and through involvement of wider healthcare team, patients and carers [15]. Whilst clinical pharmacy organisations and professional societies in different countries are known to develop and disseminate practice guidelines, there is lack of a ‘go to’ resource for societies, practitioners, and researchers in identifying all the relevant guidelines that relate to the specific areas or range of activities relevant to various clinical pharmacy services. There is a need to review scope and purpose of the published guidelines as well as assessment of quality criteria such as rigour, evidence-base and applicability of the published guidelines.

### Aim

This study aimed to review the scope of clinical pharmacy guidelines and assess the extent to which these guidelines conform to quality standards as per the AGREE II [16] instrument.

## Method

This systematic review was conducted according to the Cochrane guideline [17]. A protocol was drafted and agreed amongst the research team prior to undertaking the full review (electronic supplementary material 1).

### Eligibility criteria and study selection

Guidelines focusing on procedural activities relating to the provision of clinical pharmacy services in any health care setting were included. Guidelines published or approved by pharmacy professional societies, pharmacy regulatory organisations and best practice recommendations via special interest groups and consensus research methodology were included without any language restriction. Non-English publications were reviewed by members of the research team proficient in the language of publication. Where this was not possible, Google Translate was used for translation into English. Terminologies including ‘guideline’, ‘guidance’ or ‘practice recommendations’ as used in the document titles were included. Clinical pharmacy guidelines that focused on specific clinical area(s), such as diabetes, hypertension, or a specific patient population, such as older adults, were excluded.

### Information sources and search strategy

Medline, Embase, Guideline Central, International Pharmaceutical Abstracts and Google Scholar were searched from 2010 to January 2023. Guidelines published prior to 2010 were not considered to be representing current practices and hence excluded. Keywords and medical subject headings, where available, were searched using Boolean operators (AND, OR) to optimise the search strategy (electronic supplementary material 2). Webpages of professional societies and regulatory bodies were also searched (electronic supplementary material 3). In addition, a web-based search was undertaken using the Google advanced search functions, whereby the first 200 relevant hits were screened for eligibility.

### Selection process

The study team worked in pairs independently for title and abstract screening. The full-texts of the included articles were then screened independently by two reviewers (VP and BO). Any discrepancy or disagreements were initially resolved through discussion in pairs, and if unresolved, within the extended team. All eligible articles were transferred to EndNote 7 software for duplicates to be removed.

### Data collection process

A data extraction tool was developed using Microsoft Excel software and piloted using a sample of the included articles. The included articles were distributed amongst the reviewers (all had expertise in clinical pharmacy) who worked in pairs independently to undertake the data extraction. Data on guideline characteristics were extracted including the title, date of publication, country of published guideline, organisation approving and/or releasing the guideline and aim of the guideline. Data on the scope of the guidelines were extracted focusing on specific procedural activities covered, targeted patient populations, practice settings, health care professionals, as well as professional standards stipulated and educational and training needs of pharmacy staff. Furthermore, the study authors developed a list of items intended to assess the comprehensiveness of the guidelines and where relevant, the extent to which they supported the delivery of person-centred care, considering equity, patient safety and interprofessional collaboration. The data extraction tool was piloted and agreed between the team prior to its use.

### Quality assessment

Quality assessment of the guidelines was undertaken by independent reviewers working in pairs using the English version of the AGREE II instrument [16] after a pilot exercise within the research team. Any discrepancies were resolved through team discussions. The AGREE II instrument consists of 23 items grouped into six domains: scope and purpose; stakeholder involvement; rigour of development; clarity and presentation; applicability; and editorial independence. For each domain the allocated scores were divided by the maximum possible score to calculate the proportionate scores. A narrative synthesis of the extracted data was undertaken.

## Results

### General characteristics of eligible guidelines

**Fig. 1:**
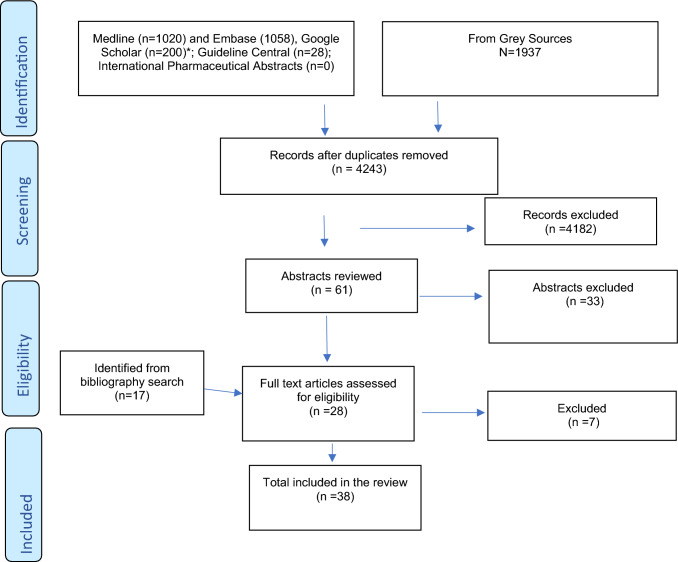
PRISMA flowchart. *First 200 titles based on relevance

Thirty-eight guidelines were included (Fig. 1 presents the PRISMA flow diagram), published between 2010 and 2022 [18–55]. Guidelines originated from Australia (n = 10), Ireland (n = 8), UK (n = 7), USA (n = 5), Netherlands (n = 3), and one from Czech Republic, Republic of Serbia, Bulgaria, Estonia, and South Africa. The majority were developed by the Society of Hospital Pharmacists of Australia (n = 9), Pharmaceutical Society of Ireland (n = 8), National Institute of Health and Care Excellence (NICE) (n = 3), and the Royal Dutch Pharmacists Association (KNMP) (n = 3). The general characteristics of the eligible guidelines are presented in Table 1.

**Table 1:** General characteristics of the included guidelines

Aim/Scope and targeted clinical pharmacy service/ activity/ process	Organization & country	Year	Professional standards stipulated	Target healthcare professional group	Target clinical setting
Practice guidelines for clinical pharmacists in South Africa to standardise quality clinical pharmacy services [18]	School of Pharmacy, Sefako Makgatho Health Sciences University, South Africa	2020	Yes	Policy makers and clinical pharmacists	All settings
Guidelines for therapeutic interchange [19]	American College of Clinical Pharmacy (ACCP), USA	2022	No	All healthcare professionals (HCPs) involved with therapeutic interchange	All settings
Guide to undertaking person-centred Inpatient, outpatient, & dispensary-based pharmacy consultations [20]	Pharmacy Dept, London NW University Hospitals Trust, UK	2019	Yes	Pharmacists involved in patient consultations	Hospital settings
Guidance for pharmacist prescribing (service, activities and procedures) [21]	General Pharmaceutical Council (GPhC), UK	2019	Yes	All pharmacy professionals competent to independent / supplementary prescribe	All settings
Guidance on the medication reconciliation process [22]	Society of Hospital Pharmacists of Australia (SHPA)	2013	Yes	Pharmacy professionals	Hospital settings
Guidance on the process for medication management and review [23]	SHPA	2013	Yes	Pharmacists	Not specifically stated
Guidance on clinical review therapeutic drug monitoring and adverse drug reaction management [24]	SHPA	2013	Yes	Pharmacists	All settings
Guidance on developing a medication management plan [25]	SHPA	2013	Yes	All HCPs	All settings
Guidance on providing medicines information [26]	SHPA	2013	Yes	Pharmacists	Hospital settings
Guidance on facilitating continuity of medication management on transition between care settings [27]	SHPA	2013	Yes	Pharmacists	All settings
Guidance on participating in interdisciplinary planning [28]	SHPA	2013	Yes	Pharmacists	Hospital settings
Guidance on how to use resources effectively in prioritising clinical pharmacy services [29]	SHPA	2013	Yes	Pharmacist	All settings
Guidance on documenting clinical activities [30]	SHPA	2013	Yes	Pharmacists	All settings
Guidelines on the disposal of medicinal products for a retail pharmacy business [31]	Pharmaceutical Society of Ireland (PSI)	2017	Yes	Retail pharmacy businesses professionals	Community pharmacy
Guidelines on counselling and medicine therapy review in the supply of prescribed medicinal products from a retail pharmacy business [32]	PSI	2019	No	Pharmacists	Community pharmacy
Guidelines on keeping records in respect of medicinal products when conducting a retail pharmacy business [33]	PSI	2019	No	Pharmacists	Community pharmacy
Guidelines on the sale or supply of non-prescription medicinal products from retail pharmacy business [34]	PSI	2018	No	Pharmacists	Community pharmacy
Guidance on the provision of testing services in pharmacies [35]	PSI	2019	No	Pharmacists	Community pharmacy
Guidance on the delivery of medicines dispensed on foot of a prescription from a retail pharmacy business [36]	PSI	2014	No	Pharmacists	Community pharmacy
Guidance on the provision of vaccination services by pharmacists in retail pharmacy businesses [37]	PSI	2022	No	Pharmacists	Community pharmacy
Guidance for pharmacists on extemporaneous dispensing [38]	PSI	2015	No	Pharmacists	Community pharmacy
Guidance on undertaking medicines optimisation activities [39]	Czech Professional Society of Clinical Pharmacists, Czech Republic	2013	Yes	Clinical pharmacists	Hospital and ambulatory care settings
Guidelines to conduct clinically oriented consultations in delivering pharmaceutical care interventions in community pharmacies [40]	Union of Pharmaceutical Associations, Serbia	2021	Yes	Community pharmacists	Community pharmacy
Guidance on good pharmaceutical practice [41]	Bulgarian Pharmaceutical Union and Ministry of Health	2020	Yes	Pharmacists	All settings
Guidelines on the processes and activities undertaken within community pharmacies [42]	National pharmaceutical society of Estonia	2021	Yes	Community pharmacists	Community pharmacy
Guideline for conducting patient consultations in community pharmacy [43]	The Dutch Royal Pharmaceutical Society	2021	Yes	Community pharmacists	Community pharmacy
Guideline for conducting clinical medication review in community pharmacy [44]	The Dutch Royal Pharmaceutical Society	2013	Yes	Community pharmacists	Community pharmacy
Guideline for conducting clinical risk management/ medication surveillance related to dispensing in community pharmacy [45]	The Dutch Royal Pharmaceutical Society	2016	Yes	Community pharmacists	Community pharmacy
Guidance on delivering comprehensive medication management (CMM) [46]	Comprehensive Medication Management in Primary Care Research Team, USA	2018	Yes	Clinical pharmacists, other HCPs, students and educators	Primary care
Guidance on patient- centred consultation skills [47]	Centre for Pharmacy Postgraduate Education, England	2019	Yes	Pharmacists and pharmacy technicians	All settings
Guidelines on dispensing practices [48]	Pharmaceutical Society of Australia	2019	Yes	Pharmacists	All settings
Guidelines on using the SBAR Tool to facilitate professional communication [49]	Institute for Healthcare Improvement, USA	2017	Yes	All HCPs	All settings
Guidelines on a patient-centred approach to support medication adherence [50]	National Institute of Clinical Excellence, (NICE) England	2019	Yes	All HCPs	All settings
Guidance on the delivery of patient-centred care [51]	Joint Commission of Pharmacy Practitioners, USA	2014	No	Pharmacists	All settings
Guidance on managing medicines in care homes [52]	NICE	2014	Yes	All HCPs who provide care in care homes	Care homes
Medicines optimisation guidelines to support the safe and effective use of medicines [53]	NICE	2014	Yes	Health and social care professionals, commissioners, providers, people taking one or more medicines, their families and carers	All settings
Medicines optimisation guidelines to help patients to make the most of medicines [54]	Royal Pharmaceutical Society, UK	2013	No	All HCPs	All settings
Comprehensive medication management guidelines [55]	Patient Centred Primary Care Collaboration, USA	2012	No	All HCPs working in primary care	All settings

The included guidelines covered a wide range of clinical pharmacy services, activities or procedures, some of which were specific to a clinical setting (e.g. primary care workplaces including community pharmacy), whereas others were applicable to a range of clinical settings. The included guidelines provided limited details on resources required for implementation of the guidelines. Two exceptions were guidelines published by the Pharmaceutical Society of Ireland, namely guidance on the provision of testing services in community pharmacies [35], and guidance on the provision of vaccination services in the community pharmacy setting [37]. Both guidelines provided details of facilities and equipment, the need for public communication, and resources to support quality delivery of services.

To underpin recommendations, the majority of guidelines (n = 34) made reference to either nationally published professional standards, such as those published by the national pharmacy professional body, such as the UK General Pharmaceutical Council [21] and the Society of Hospital Pharmacists of Australia [22–30], or professional standards published by national institutes or organisations concerned with optimising the delivery of health care, such as NICE [50, 52, 53].

### Extent to which guidelines supported delivery of person-centred care

Most guidelines (n = 25) encouraged patient involvement in decision-making, and included specific guidance on effective patient communication (n = 23). However, exceptions included six guidelines published between 2012 and 2013 [24, 28–30, 39, 44]; and guidelines issued in countries where clinical pharmacy services were described to be in early development phases [18, 39, 41, 42]. Most of the guidelines (n = 22) stated the importance of involving patients´ family and carers during the process of clinical pharmacy service provision [19, 21, 22, 24–27, 30, 32, 34–38, 40, 41, 43, 44, 50, 52–54].

In terms of ensuring equity and inclusivity in services delivery, five of the guidelines articulated the need to provide culturally sensitive information to patients [21, 43, 50, 52, 55], and seven included consideration for people with physical, sensory or learning disabilities [21, 35, 37, 43, 45, 50, 52]. The majority of these guidelines were published after 2016, and by bodies in the UK [21, 50, 52], Ireland [35, 37] and the Netherlands [43, 45].

An assessment of eligible guidelines for person-centeredness is presented in Table 2.

**Table 2:** Assessment of the included guidelines for person-centeredness

Guideline	Advocates for patient involvement in decision making	Includes recommendations for effective communication	Advocates for multidisciplinary working	Advocates for referral to other services	Advocates for patient monitoring and follow up	Advocates for patient safety	Advocates for involving family and carers	Advocates for providing culturally sensitive information	Includes consideration for people with physical, sensory or learning disabilities
[18]	No	Yes	Yes	No	Yes	Yes	No	No	No
[19]	Yes	Yes	Yes	No	No	Yes	Yes	No	No
[20]	No	Yes	No	No	No	Yes	No	No	No
[21]	Yes	Yes	Yes	Yes	Yes	Yes	Yes	Yes	Yes
[22]	Yes	Yes	Yes	Yes	Yes	Yes	Yes	No	In part
[23]	Yes	In part	Yes	Yes	Yes	Yes	No	No	No
[24]	No	No	Yes	Yes	Yes	Yes	Yes	No	No*
[25]	Yes	No	Yes	Yes	Yes	No	Yes	No	No
[26]	Yes	Yes	Yes	Yes	Yes	No	Yes	No	No
[27]	Yes	Yes	Yes	Yes	Yes	No	Yes	No	No
[28]	No	No	Yes	Yes	No	No	No	No	No
[29]	No	No	No	Yes	No	Yes	No	No	No
[30]	No	No	Yes	No	No	Yes	Yes	No	No
[31]	N/a	In part	In part	N/a	N/a	Yes	No	No	No
[32]	Yes	Yes	N/a	Yes	Yes	Yes	Yes	Yes	No
[33]	N/a	N/a	N/a	N/a	N/a	Yes	N/a	N/a	N/a
[34]	Yes	Yes	N/a	Yes	No	Yes	Yes	Yes	No
[35]	Yes	Yes	N/a	Yes	Yes	Yes	Yes	No	Yes
[36]	Yes	Yes	N/a	No	No	Yes	Yes	No	No
[37]	Yes	Yes	Yes	Yes	Yes	Yes	Yes	Yes	Yes
[38]	Yes	No	N/a	No	No	Yes	Yes	No	No
[39]	No	No	Yes	Yes	Yes	Yes	No	No	No
[40]	Yes	Yes	Yes	Yes	Yes	Yes	Yes	No	No
[41]	No	No	Yes	Yes	Yes	Yes	Yes	No	No
[42]	No	No	Yes	Yes	No	Yes	No	No	No
[43]	Yes	Yes	Yes	Yes	Yes	Yes	Yes	Yes	Yes
[44]	No	No	Yes	No	Yes	Yes	Yes	No	No
[45]	No	No	Yes	No	Yes	Yes	No	No	Yes
[46]	Yes	Yes	Yes	Yes	Yes	Yes	No	No	No
[47]	Yes	Yes	Yes	Yes	Yes	Yes	No	No	No
[48]	Yes	Yes	Yes	Yes	Yes	Yes	No	In part	In part
[49]	Yes	No	Yes	Yes	Yes	Yes	No	No	No
[50]	Yes	Yes	Yes	Yes	Yes	Yes	Yes	Yes	Yes
[51]	Yes	No	Yes	Yes	Yes	Yes	No	No	No
[52]	Yes	Yes	Yes	Yes	Yes	Yes	Yes	Yes	Yes
[53]	Yes	Yes	Yes	Yes	Yes	Yes	Yes	No	No
[54]	Yes	No	Yes	No	Yes	Yes	Yes	No	No
[55]	Yes	Yes	Yes	Yes	Yes	Yes	No	Yes	No

(*Includes reference made to other guidelines)

### AGREE II scores

**Fig. 2:**
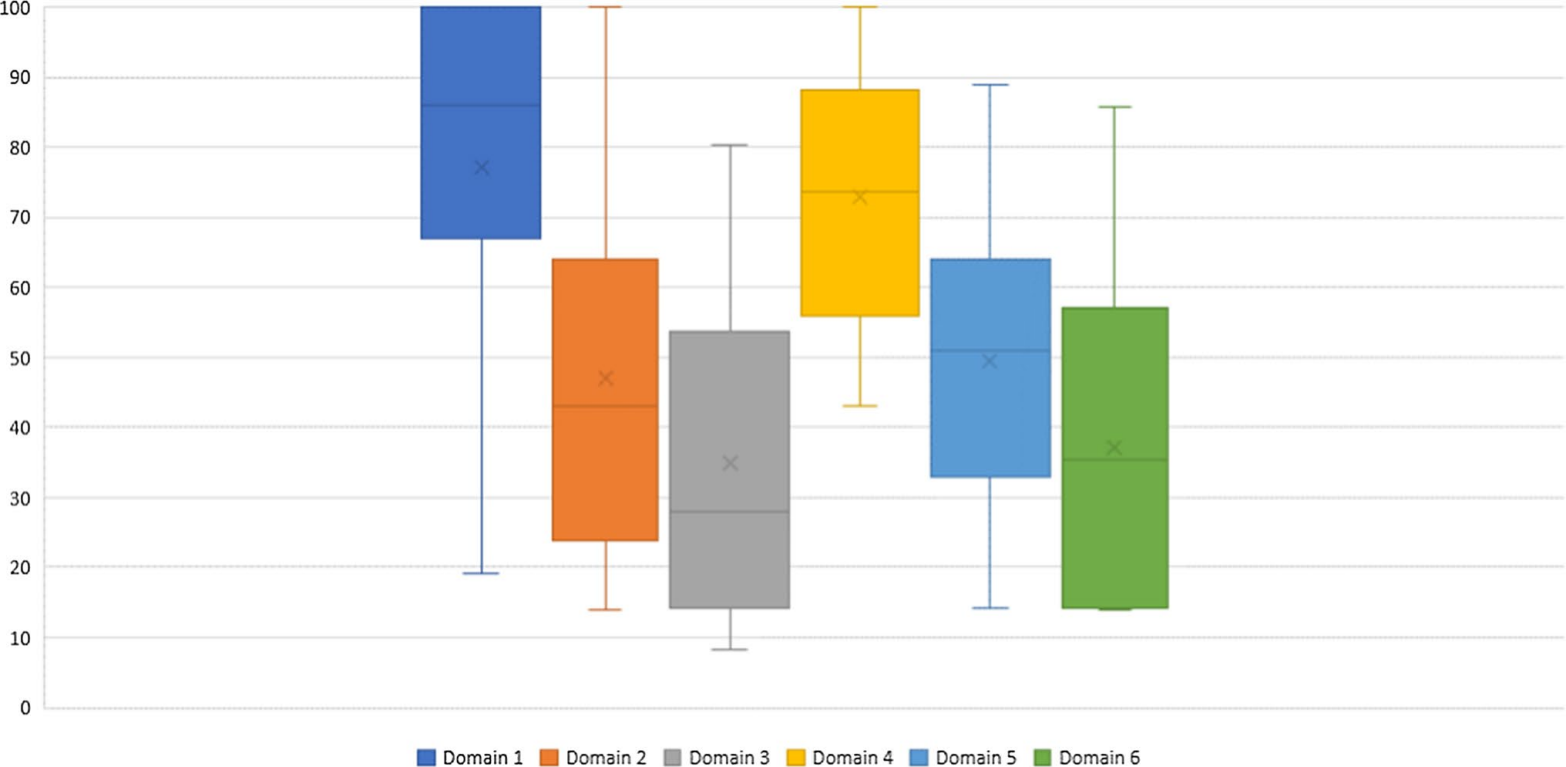
Scores obtained from each domain of AGREE II tool. Domain 1: scope and purpose; Domain 2: stakeholder involvement; Domain 3: rigour of development; Domain 4: clarity of presentation; Domain 5: applicability; Domain 6: editorial independence

Among the domains of the AGREE II instrument, the highest score was for Domain 1: *Scope and purpose,* and the lowest for Domain 3: *Rigour of development.* Table 3 presents the AGREE II scores for each domain and the cumulative totals for each of the included guidelines; and scores obtained for each domain of the AGREE II instrument are displayed in Fig. 2.

**Table 3:** Results from AGREE-II Instrument

Guideline	Domain 1 (%)	Domain 2 (%)	Domain 3 (%)	Domain 4 (%)	Domain 5 (%)	Domain 6 (%)	Overall quality (1–7)	Recommendation of use
[18]	71.0	24.0	18.0	71.0	42.0	79.0	4	Recommended with modification
[19]	47.0	43.0	45.0	86.0	36.0	79.0	4	Recommended with modification
[20]	38.0	43.0	14.0	57.0	25.0	14.0	2	Recommended with modification
[21]	100.0	66.7	33.3	83.3	52.1	41.7	6	Recommended with modification
[22]	80.0	24.0	32.0	80.0	52.0	35.0	5	Recommended with modification
[23]	86.0	29.0	34.0	100.0	79.0	57.0	5	Recommended with modification
[24]	94.4	52.8	24.0	86.1	56.3	29.2	5	Recommended with modification
[25]	38.1	28.6	18.4	71.4	25.0	14.3	2	Not recommended
[26]	38.1	23.8	14.3	66.7	23.8	14.3	2	Not recommended
[27]	38.1	21.4	16.1	76.2	17.8	14.3	2	Not recommended
[28]	38.1	23.8	14.3	61.9	14.3	14.3	2	Not recommended
[29]	19.0	19.0	14.3	61.9	14.3	14.3	2	Not recommended
[30]	57.1	33.3	14.3	76.2	23.8	14.3	2	Not recommended
[31]	100.0	57.0	38.0	100.0	86.0	57.0	6	Recommended
[32]	86.0	14.0	14.0	48.0	50.0	14.0	3	Recommended with modification
[33]	67.0	14.0	14.0	52.0	50.0	14.0	3	Recommended with modification
[34]	86.0	14.0	14.0	52.0	50.0	14.0	3	Recommended with modification
[35]	86.0	14.0	14.0	48.0	64.0	14.0	3	Recommended with modification
[36]	71.0	14.0	14.0	48.0	54.0	14.0	3	Recommended with modification
[37]	100.0	43.0	14.0	48.0	61.0	14.0	4	Recommended
[38]	86.0	14.0	14.0	43.0	50.0	14.0	3	Recommended
[39]	94.4	63.9	24.0	94.4	64.6	29.2	6	Recommended with modification
[40]	100	63.9	53.1	94.4	41.7	41.7	6	Recommended with modification
[41]	72.2	38.9	8.3	44.4	20.8	25.0	2	Not recommended
[42]	88.9	66.7	14.6	52.8	39.6	25.0	5	Not recommended
[43]	90.0	86.0	65.0	100.0	64.0	52.0	6	Recommended with modification
[44]	71.0	57.0	57.0	66.0	40.0	36.0	5	Recommended with modification
[45]	86.0	52.0	60.0	84.0	25.0	36.0	5	Recommended with modification
[46]	90.0	54.0	54.0	100.0	64.0	57.0	6	Recommended
[47]	100.0	57.0	68.0	100.0	89.0	57.0	6	Recommended
[48]	100.0	100.0	73.0	100.0	79.0	57.0	6	Recommended
[49]	100.0	43.0	46.0	100.0	79.0	57.0	6	Recommended
[50]	100.0	100.0	78.0	76.0	53.5	64.0	6	Recommended with modification
[51]	66.6	52.3	37.5	57.1	61.9	47.6	3	Not recommended
[52]	100	100	77	85.7	64.2	85.7	6	Recommended
[53]	100	100	80.4	76.1	60.7	64.3	6	Recommended
[54]	76.1	52.3	53.5	57.1	35.7	57.1	4	Recommended with modification
[55]	71.4	80.9	48.2	66.6	75.0	47.6	4	Recommended with modification

Domain 1: Scope and purpose; Domain 2: Stakeholder involvement; Domain 3: Rigour of development; Domain 4: Clarity of presentation; Domain 5: Applicability; Domain 6: Editorial independence

A total of ten of the included guidelines scored 100% for Domain 1 [21, 31, 37, 40, 47–50, 52, 53]; other guidelines which did not score 100% were either lacking details relating to the intended target population, such as age, co-morbidities or excluded populations; or the overall objective of the guideline was poorly defined. Four guidelines (one developed by Pharmaceutical Society of Australia [48], and three developed by NICE [50, 52, 53]) scored 100% for Domain 2: *Stakeholder involvement*. Many of the guidelines scored poorly in this domain; most frequently there were insufficient details to ascertain who the stakeholders involved in the development process were and how their views were considered in the development of the guideline (Table 3). For Domain 3*: Rigour of development*, no guidelines scored 100%. Guidelines lacked details pertaining to the search strategy employed to collate the cited evidence; strengths and limitations of the included evidence; methods for formulating the recommendations; and processes adopted for external review and update the guideline. *Clarity of presentation* (Domain 4): Seven guidelines scored 100% [23, 31, 43, 46–49], indicating that key recommendations were easy to identify and interpret from the guidelines. For Domain 5: *Applicability*, no guidelines scored 100%. The guidelines failed to comprehensively describe the barriers and facilitators to application, including the resource implications; and did not provide adequate details regarding monitoring criteria to measure application of the guideline recommendations. For *Editorial independence* (Domain 6), no guidelines scored 100%, either due to the absence of an explicit statement to describe contributing stakeholders’ conflicts of interest (if any) or failure to report the funding body’s influence on the content of the guideline (where relevant).

Overall, 9 out of 38 guidelines were recommended without modification for use in practice based on the AGREE-II instrument [31, 37, 38, 46–49, 52, 53].

## Discussion

### Key findings

The majority of guidelines represented a limited number of countries including Australia, Ireland, UK and USA, and described specific clinical pharmacy services or activities. While greater focus was on aspects such as medication review and medication reconciliation, there was little attention paid to education, training and competency development, which are central to the acquisition of these skills, for development of new services and to encourage advanced practice. Most of the guidelines promoted multidisciplinary working which underlines the pharmacy profession’s approach to improving the use of medicines through collaboration with other healthcare professionals.

### Interpretation

The content and focus of most of the guidelines related to services such as medication review, medicines reconciliation and medication management including provision of dispensing services and clinical checking. Most of the official bodies approving and releasing the guidelines were professional regulators, professional society bodies, Health Technology Assessment bodies and independent healthcare bodies. Only a small number of guidelines focused on person-centred care and clinical communication. There is scope to develop international guidelines that can assist best practices in the delivery of person-centred care and clinical communications considering the relevance of these activities to the range of clinical pharmacy services and potential for application across diverse settings and countries.

Equity and patient-centred care are important aspects of healthcare, particularly at a time when migration and displacement of population groups has created multi-ethnic societies all around the world, and ageing populations are leading to an increasing proportion of citizens dependent upon health and social care services. The results identified that while 20 of the 38 guidelines endorsed the involvement of family and carers, only a few emphasised on providing culturally sensitive information (n = 4) or consideration of people with physical, sensory, or learning disabilities (n = 6). Allied to this, only a minority (n = 6) addressed applicability which assesses implementation and monitoring. This finding strongly suggests that a stronger vision and urgency is needed to support practice implementation of published guidelines.

Using the AGREE II tool, the quality of the guidelines was found to be low to moderate. Across the guidelines, scope and clarity aspects of the guidelines were rated higher than rigour of development, stakeholder involvement and applicability. For example, only a few demonstrated a systematic, evidence-based approach to their recommendations which is surprising given that most were produced by regulators or professional representative bodies. The extent of stakeholder involvement in the development process were unclear in most guidelines. Similarly, the low scores for the rigour of development and editorial independence domains were notable. Although just over half (n = 20) of the guidelines were published between 5 to 10 years ago, all but one of the others were less than 5 years old. Over this period the adoption of systematic and evidence-based methods of guideline development have been accepted as best practice and the AGREE II instrument has been extensively used since 2009 [56].

The role of the pharmacist and the place of clinical pharmacy services remain contested facets of healthcare in many countries [5, 57], and without rigorous, evidence-based guidelines, clinical pharmacy development is likely to continue to struggle to gain more widespread recognition. To remedy this, guideline development bodies, including professional societies that develop clinical practice guidelines should focus efforts on the quality aspects of guideline development and resources to support implementation. Utilisation of skilled professionals and strengthening the clinical pharmacy support staff team is key to promote safe and effective use of medications and provide person-centred care [58]. At the same time, guidelines should also be able to carefully consider practical challenges for practitioners and administrators and how to implement recommendations in a resource-constrained environment. Quality guidelines should be better utilised in various languages and in versions adjusted to the local situation and needs in different countries.

### Strengths and limitations

This is the first systematic review published on scope and quality of clinical pharmacy guidelines. It encompassed guidelines published in different countries and used the validated AGREE II instrument to assess the quality of the included guidelines. The study reviewers used a previously defined approach for quality assessment of guidelines, reviewed texts independently and thoroughly, discussed their approach, and resolved any difficulties encountered during the process in these discussions. However, inter-rator agreement was not assessed using statistical approaches and some divergence in approach may have remained.

Limitations of the AGREE II instrument have been previously discussed in the literature. The six domains in the AGREE II instrument are independent of each other and the tool does not allow calculation of a single global score based on domain scores [59]. It is also worth noting that from the perspective of guideline development bodies, some of the expectations laid out by the AGREE II criteria require extensive resources to implement compared to others. For example, satisfying the appraisal criteria around ‘rigour of development’ (domain 3) requires guideline development bodies to undertake a rigorous systematic review of existing literature prior to formulating the guidelines, whereas, satisfying domain criteria around ‘clarity of presentation’ and ‘editorial independence’ could be argued to be relatively less resource intensive.

### Future research

Reviewing guidelines specific to a clinical condition, technology or patient population was not within the scope of this study. Future research should evaluate published guidelines in specific areas of practice regarding their scope, strengths, limitations and applicability. Pharmacists’ roles are increasing internationally with emphasis on delivery of cognitive services and independent prescribing [6, 60–62]. There is an opportunity for international professional practice societies and health systems to make a positive impact on patient care globally by developing common practice guidelines focusing on core pharmacy practice activities. Such guidelines could be adapted further by different nations and geographies for the recommendations to be implemented in local/national contexts.

## Conclusion

Clinical pharmacy guidelines included in this review represent a limited number of countries, settings and services. There is a scope to co-develop and disseminate internationally applicable guidelines in promoting person-centred care and clinical communication given their relevance to a range of clinical pharmacy services, settings, and countries. International best practice guidelines for various clinical pharmacy activities may provide a basis for the development of country-specific guidelines and clinical pharmacy services in different countries and healthcare systems including low and middle income countries. Quality of most guidelines as assessed by the AGREE II instrument was found to be low to moderate. Developers of future clinical pharmacy guidelines need to focus more on all quality domains and should adopt a systematic approach to guideline development to generate evidence supporting establishment of modern clinical pharmacy services in different countries, helping to improve healthcare quality.

### Supplementary Information

Below is the link to the electronic supplementary material.

Supplementary file1 (DOCX 19 kb)

Supplementary file2 (DOCX 12 kb)

Supplementary file3 (DOCX 26 kb)
