# Supplementary material for: Scope, content and quality of clinical pharmacy practice guidelines: a systematic review
Source: Int J Clin Pharm. 2023 Nov 22;46(1):56–69. doi: 10.1007/s11096-023-01658-x (PMC10830799; doi:10.1007/s11096-023-01658-x)
Supplement: Supplementary file 1 — Supplementary file1 (DOCX 19 kb) [file 11096_2023_1658_MOESM1_ESM.docx]

**STUDY PROTOCOL**

**Title: Scope and quality of guidelines to inform clinical pharmacy practice activities: a systematic review**

**Review question**

1. What is the quality and scope of clinical pharmacy practice guidelines in terms of specific procedural activities covered, practice settings, pharmacy personnel targeted, professional standards stipulated, educational and training needs of pharmacy staff and patient populations?
2. How well do the clinical pharmacy practice guidelines conform to the quality standards as per the AGREE II instrument? (AGREE II is the international tool to assess the quality and reporting of practice guidelines).

# Searches

We will use the following electronic database: MEDLINE, Embase, PsycINFO, Guideline Central, Cochrane Library, Google, Google Scholar and Opengrey.eu. Search will be restricted from year 2010 to current. We will use keywords and medical subject headings where available to identify guidelines in clinical pharmacy practice. Boolean operators (AND, OR) will be used. We will also search guidelines through webpages of professional societies and regulatory bodies.

# Types of study to be included

Practice guidelines focused on procedural activities related to the provision of clinical pharmacy services will be included. While the identification the scope of the guidelines is one of the objectives, activities focused on the guidelines relevant for inclusion in this review could include counselling and communication practices, medicines reviews and reconciliations and drug history taking. Guidelines published published/approved by pharmacy professional societies, pharmacy regulatory organisations and best practice recommendations via special interest groups and consensus research methodology will be included. Guidelines focused on any setting of health care such as hospital, primary care and the community will be included.

We will exclude guidelines focused on specific clinical area(s) (such as antimicrobial stewardship) or a specific patient population (e.g. geriatrics).

# Condition or domain being studied

General

# Intervention(s), exposure(s)

Guidelines to inform clinical pharmacy practice activities.

# Comparator(s)/control

None

# Context

Any clinical setting will be included.

# Main outcome(s)

Scope of the guidelines in terms of: the areas of clinical pharmacy practice procedure/ activities covered; clinical settings, geographies and patient populations; educational and training needs of pharmacy staff stipulated and quality as per the AGREE II standards?

## *Measures of effect*

Not applicable.

# Additional outcome(s)

None.

## *Measures of effect*

Not applicable.

# Data extraction (selection and coding)

This review will be reported in accordance with PRISMA guidelines. The titles and abstracts will be screened to identify potentially eligible guidelines. All identified guidelines will be screened against eligibility criteria

The reviewers will screen search outputs, identify eligible guidelines and extract data. Any discrepancy or disagreement will be resolved through further discussion. Full text will be screened by reviewers to identify guidelines that will be included for data extraction. Data will be extracted using a spreadsheet of Microsoft Excel®.

Extracted data will include: title, date of publication, country of published guideline, organization and, aims and scope of the guidelines, resources needed to implement the guidelines, professional standards described including workforce, leadership and systems governance.

# Risk of bias (quality) assessment

Guidelines included in this review will be assessed by using the English version of the AGREE II instrument. It consists of 23 items grouped in six domains: scope and purpose, stakeholder involvement, rigor of development, clarity and presentation, applicability, and editorial independence. At least 2 independent reviewers (native speakers) will review in a particular language relevant guidelines and possible discrepancies will be resolved in discussions and in agreement with the third independent reviewer (also a native speaker).

# Strategy for data synthesis

A narrative synthesis (or meta-synthesis, if possible) of the data will be undertaken to answer study aim. Data will be summarized and presented using data summary tables and narrated paragraphs.

# Analysis of subgroups or subsets

Not applicable.

# Contact details for further information

Vibhu Paudyal

v.paudyal@bham.ac.uk

# Review team members and their organisational affiliations

All research committee members plus Derek Stewart (will be listed)

# Type and method of review

Systematic review
