# Supplementary material for: Scope, content and quality of clinical pharmacy practice guidelines: a systematic review
Source: Int J Clin Pharm. 2023 Nov 22;46(1):56–69. doi: 10.1007/s11096-023-01658-x (PMC10830799; doi:10.1007/s11096-023-01658-x)
Supplement: Supplementary file 2 — Supplementary file2 (DOCX 12 kb) [file 11096_2023_1658_MOESM2_ESM.docx]

Search strategy example: MEDLINE

1 pharmacist.mp. or Pharmacists/ (26844)

2 Education, Pharmacy/ or Pharmacy/ or pharmacy.mp. or Community Pharmacy Services/ or Pharmacy Service, Hospital/ or Pharmacy Research/ or Pharmacy Administration/ (63502)

3 (clinical pharmac* or clinical pharmacist*).mp. [mp=title, abstract, original title, name of substance word, subject heading word, floating sub-heading word, keyword heading word, organism supplementary concept word, protocol supplementary concept word, rare disease supplementary concept word, unique identifier, synonyms] (17020)

4 (drug utilisation review* or medication review* or pharmaceutical care* or home medicines review* or medicines reconcilliation or Medication therapy management or drug regimen review).mp. [mp=title, abstract, original title, name of substance word, subject heading word, floating sub-heading word, keyword heading word, organism supplementary concept word, protocol supplementary concept word, rare disease supplementary concept word, unique identifier, synonyms] (6688)

5 counselling.mp. or Counseling/ (60144)

6 communication.mp. or Communication/ (361424)

7 1 or 2 or 3 (86648)

8 4 or 5 or 6 (423911)

9 Guideline/ or Practice Guideline/ (36477)

10 guideline*.mp. (486936)

11 Practice Guidelines as Topic/ or recommendation*.mp. (370645)

12 consensus.mp. or Consensus/ (182213)

13 9 or 10 or 11 or 12 (817829)

14 7 and 8 and 13 (1485)

15 limit 14 to (english language and yr="2010 -Current") (1020)
