# Supplementary material for: Scope, content and quality of clinical pharmacy practice guidelines: a systematic review
Source: Int J Clin Pharm. 2023 Nov 22;46(1):56–69. doi: 10.1007/s11096-023-01658-x (PMC10830799; doi:10.1007/s11096-023-01658-x)
Supplement: Supplementary file 3 — Supplementary file3 (DOCX 26 kb) [file 11096_2023_1658_MOESM3_ESM.docx]

**Professional societies and other grey resources searched to obtain databases**

| **Organisation** |
| --- |
| European Society of Clinical Pharmacy |
| The International Pharmaceutical Federation (FIP) |
| European Association of Hospital Pharmacy |
| American Pharmacist Association |
| American College of Clinical Pharmacy |
| American Society of Health System Pharmacy |
| Pharmaceutical Group of the European Union |
| Pharmaceutical Society of New Zealand |
| Pharmaceutical Society of Ireland |
| Pharmaceutical Society of Northern Ireland |
| Chinese Pharmaceutical Association |
| Australasian college of Pharmacy |
| Canadian Pharmacists Association |
| General Pharmaceutical Council |
| Royal Pharmaceutical Society (GB) |
| Malta medicines authority |
| Malta chamber of pharmacists |
| MAHP - Malta Association of Hospital Pharmacists |
| Pharmacy Professional Society of Germany |
| Pharmacy Professional Society of Austria |
| Pharmacy Professional Society of Czech Republic |
| Pharmacy Professional Society of Croatia |
| Pharmacy Professional Society of Republic of Serbia |
| Pharmacy Professional Society of Bulgaria |
| Pharmacy Professional Society of Estonia |
| Pharmacy Professional Society of Netherlands |
| Pharmacy Professional Society of Turkey |
| Pharmacy Professional Society of Switzerland |
| Pharmacy Professional Society of Slovakia |

****INCLUDED**

- [Guidelines on the Disposal of Medicinal Products for a Retail Pharmacy Business (Version 3, Oct. 2017)](http://www.thepsi.ie/Libraries/Folder_Pharmacy_Practice_Guidance/01_5_Disposal_of_Medicinal_Products_for_Retail.sflb.ashx)
- [Guidelines on Counselling and Medicine Therapy Review in the Supply of Prescribed Medicinal Products from a Retail Pharmacy Business (Version 4, October 2019)](http://www.thepsi.ie/Libraries/Folder_Pharmacy_Practice_Guidance/01_11_Guidelines_on_Counselling_and_Medicine_Therapy_Review_in_the_Supply_of_Prescribed_Medicinal_Products.sflb.ashx)
- [Guidelines on Keeping Records in Respect of Medicinal Products when Conducting a Retail Pharmacy Business (Version 3, April 2019)](https://www.thepsi.ie/Libraries/Folder_Pharmacy_Practice_Guidance/1_11_Guidelines_on_the_Keeping_of_Records.sflb.ashx)
- [Guidelines on the Sale or Supply of Non-Prescription Medicinal Products from a Retail Pharmacy Business (Version 1, November 2018)](https://www.thepsi.ie/Libraries/Folder_Pharmacy_Practice_Guidance/01_11_Guidelines_on_Non-Prescription_Medicines.sflb.ashx)
- [Guidance on the Provision of Testing Services in Pharmacies (Version 3, October 2019)](http://www.thepsi.ie/Libraries/Folder_Pharmacy_Practice_Guidance/PPGF_02_3_Provision_of_Testing_Services_in_Pharmacies.sflb.ashx)
- [Guidance on the Delivery of Medicines Dispensed on foot of a Prescription from a Retail Pharmacy Business (Version 1, July 2014)](http://www.thepsi.ie/Libraries/Folder_Pharmacy_Practice_Guidance/PPGF_2_5_Delivery_of_Medicines.sflb.ashx)
- [Guidance on the Provision of Vaccination Services by Pharmacists in Retail Pharmacy Businesses (Version 7, October 2021)](https://thepsi.ie/Libraries/Folder_Pharmacy_Practice_Guidance/2_4_Guidance_on_the_Provision_of_Vaccination_Services_by_Pharmacists_in_a_Retail_Pharmacy_Businesses.sflb.ashx)
- [Guidance for Pharmacists on Extemporaneous Dispensing (Version 1, June 2015)](http://www.thepsi.ie/Libraries/Folder_Pharmacy_Practice_Guidance/02_Guidance_for_Pharmacists_on_Extemporaneous_Dispensing_V1_0.sflb.ashx)

<https://www.pharmacyregulation.org/content/practice-guidance-pharmacist-prescribers-february-2020>

https://www.shpa.org.au/publications-resources/standards-of-practice/standards-of-practice-for-clinical-pharmacy-services

we have downloaded individual chapters of these from the journal resources here <https://onlinelibrary.wiley.com/toc/20552335/2013/43/S2> Al included are in the relevant folder

1

Gregorová J.: METODIKA I: PRÁCE NA ODDĚLENÍ KLINICKÉ FARMACIE, OPTIMALIZACE FARMAKOTERAPIE PACIENTA (Methodology I: The Work on Clinical Pharmacy Department, Optimization of the Patient’s Pharmacotherapy), AMCA, Prague 2013. Available online at: <https://www.coskf.cz/spolecnost/metodiky-a-koncepce>

1

Branislava Miljkovic, Sandra Vezmar Kovacevic, Katarina Vucicevic, Marija Jovanovic, Milena Kovacevic, Milica Culafic. The guidelines for pharmacists in primary health care. Faculty of Pharmacy, University of Belgrade and Pharmaceutical Association of Serbia. Belgrade, 2021. Available at: https://www.zdravlje.gov.rs/view_file.php?file_id=2268&cache=sr

1

Раздел VII. Клиничен магистър-фармацевт . ПРАВИЛА ЗА ДОБРА ФАРМАЦЕВТИЧНА ПРАКТИКА. (Section VII. Clinical pharmacist. Good pharmacy practice guidelines). Clinical Pharmacy Association in Bulgaria. Jan 2020; page 18. Available at: https://www.bphu.bg/upload/files/Pravila_Good%20Pharmacy%20Practice-VERSION–2020.pdf

1

Alamaa Aas K, Entsik-Grünberg T, Israel M, Jürgenson S, Kiloman M, Laius O, Markov M, Monvelt H, Pedosk L-K, Petratškov K, Roolaid S, Rootslane L, Ruuben L, Sarv K, Sepp K, Talve T, Tuula A, Uibokand P, Volmer D. Community Pharmacy Services Quality Guidelines – CPSQG (2012, updated in 2016 and 2021). National pharmaceutical society of Estonia. 2021. Available at: Apteegiteenuse kvaliteedijuhis 2021 (veeb).cdr (efs.ee)

**1-KNMP guideline Consultation**

[[Consultation | KNMP](https://www.knmp.nl/richtlijnen/consultvoering)]

**2-KNMP guideline Medication assessment**

[Medication assessment | Knmp](https://www.knmp.nl/richtlijnen/medicatiebeoordeling)

**3-KNMP guideline Medication monitoring**

[Medication monitoring | Knmp](https://www.knmp.nl/richtlijnen/medicatiebewaking)

**EMBASE**

1- Bronkhorst E, Gous AGS, Schellack N. Practice Guidelines for Clinical Pharmacists in Middle to Low Income Countries. Front Pharmacol. 2020 Jun 30;11:978. doi: 10.3389/fphar.2020.00978.

**GOOGLE SCHOLAR**

1- Guidelines for therapeutic interchange. American College of Clinical Pharmacy. Pharmacotherapy. 1993 May-Jun;13(3):252-6. PMID: 8321738.

**MEDLINE**

1- Barnett NL. Guide to undertaking person-centred inpatient (ward) outpatient (clinic) and dispensary-based pharmacy consultations. Eur J Hosp Pharm. 2020 Sep;27(5):302-305. doi: 10.1136/ejhpharm-2018-001708.
